# Signaling mechanisms of SARS-CoV-2 Nucleocapsid protein in viral infection, cell death and inflammation

**DOI:** 10.7150/ijbs.72663

**Published:** 2022-07-11

**Authors:** Wenbiao Wang, Junzhe Chen, Xueqing Yu, Hui-Yao Lan

**Affiliations:** 1 Medical Research Center and Guangdong-Hong Kong Joint Laboratory for Immunity and Genetics of Chronic Kidney Disease, Guangdong Provincial People's Hospital, Guangdong Academy of Medical Sciences, Guangzhou, China.; 2 Department of Nephrology, The Third Affiliated hospital, Southern Medical University, Guangzhou, China.; 3 Departments of Medicine & Therapeutics, Li Ka Shing Institute of Health Sciences, and Lui Che Woo Institute of Innovative Medicine, The Chinese University of Hong Kong, Hong Kong, China.; 4 The Chinese University of Hong Kong-Guangdong Academy of Sciences/Guangdong Provincial People's Hospital Joint Research Laboratory on Immunological and Genetic Kidney Diseases, The Chinese University of Hong Kong, Hong Kong, China.

## Abstract

COVID-19 which is caused by severe acute respiratory syndrome coronavirus (SARS-CoV-2) has posed a worldwide pandemic and a major global public health threat. SARS-CoV-2 Nucleocapsid (N) protein plays a critical role in multiple steps of the viral life cycle and participates in viral replication, transcription, and assembly. The primary roles of N protein are to assemble with genomic RNA into the viral RNA-protein (vRNP) complex and to localize to the replication transcription complexes (RTCs) to enhance viral replication and transcription. N protein can also undergo liquid-liquid phase separation (LLPS) with viral genome RNA and inhibit stress granules to facilitate viral replication and assembly. Besides the function in viral life cycle, N protein can bind GSDMD to antagonize pyroptosis but promotes cell death via the Smad3-dependent G1 cell cycle arrest mechanism. In innate immune system, N protein inhibits IFN-β production and RNAi pathway for virus survival. However, it can induce expression of proinflammatory cytokines by activating NF-κB signaling and NLRP3 inflammasome, resulting in cytokine storms. In this review article, we are focusing on the signaling mechanisms of SARS-CoV-2 N protein in viral replication, cell death and inflammation.

## 1. Life cycle of SARS-CoV-2

**Figure 1:**
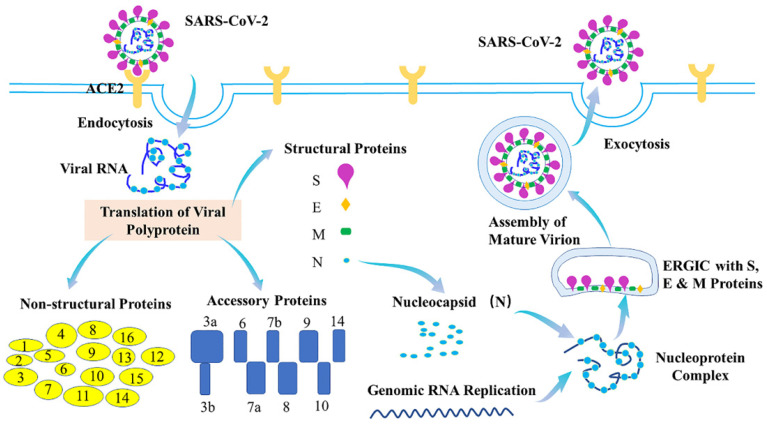
Life cycle of SARS-CoV-2. During the viral infection, SARS-CoV-2 S protein binds to ACE2 to inject its genome into host cell via endocytosis. The viral genome comprises 14 ORFs, encoding 16 Nsps, 9 ORF proteins and 4 structural proteins. Nucleocapsid (N) protein binds to viral genome RNA into ribonucleoprotein (RNP) complex, assisting membrane (M) and envelope (E) proteins to initiate viral assembly. The assembly of mature SARS-CoV-2 virions-containing vesicles which occurs within the ERGIC can fuse with the plasma membrane during exocytosis and release SARS-CoV-2 into the extracellular space.

The coronavirus disease 2019 (COVID-19) is caused by severe acute respiratory syndrome coronavirus (SARS-CoV-2). With causing more than 518 million confirmed cases and more than 6.25 million deaths worldwide (World Health Organization (WHO)), COVID-19 has resulted in public health crises and widespread economic disruption. Typical clinical symptoms of COVID-19 are fever, dry cough, fatigue and shortness of breath, while severe patients may progress to acute respiratory distress syndrome (ARDS), acute lung injury, septic shock, or even death 1-4. SARS-CoV-2 with ~30kb viral genome RNA is an enveloped, positive-stranded RNA virus which belongs to the β-coronaviruses. SARS-CoV-2 genome consists of 14 functional open reading frames (ORFs), including two regions (ORF1a and ORF1b) for 16 non-structural proteins (Nsp1-Nsp16), nine regions for nine putative accessory proteins, and other regions for four structural proteins, spike (S), envelope (E), membrane (M), and nucleocapsid (N) proteins 5-7. Of them, SARS-CoV-2 S protein binds to its cellular receptor, angiotensin-converting enzyme 2 (ACE2) 8,9, to enter the cells. Additionally, the host serine protease TMPRSS2 is important for priming of the S protein for receptor interactions and entry 9. Many host proteins can also function as cofactors for viral entry, such as heparin sulfate proteoglycans, C-type lectins, neuropilin-1 and furin 10-13. After being entry, the viral and host membranes can fuse together and then release the positive sense, single-stranded RNA genome of SARS-CoV-2 that directly translates into the structural and nonstructural proteins 9. N protein can also bind to the viral genome RNA to form the ribonucleoprotein (RNP) complex, while the M and E proteins can initiate the viral assembly 14,15. The 16 nonstructural proteins (Nsps) can facilitate formation of the viral replication-transcription complex 16,17 and promote the traffic to the ER or Golgi membranes. In addition, they can combine with genomic RNA and N proteins to create nascent viral particles. Occurring within the ER-to-Golgi intermediate compartment (ERGIC), the assembly of mature SARS-CoV-2 virions-containing vesicles can fuse with the plasma membrane during exocytosis and release SARS-CoV-2 into the extracellular space 18,19 (Figure 1).

## 2. SARS-CoV-2 Nucleocapsid protein

**Figure 2:**
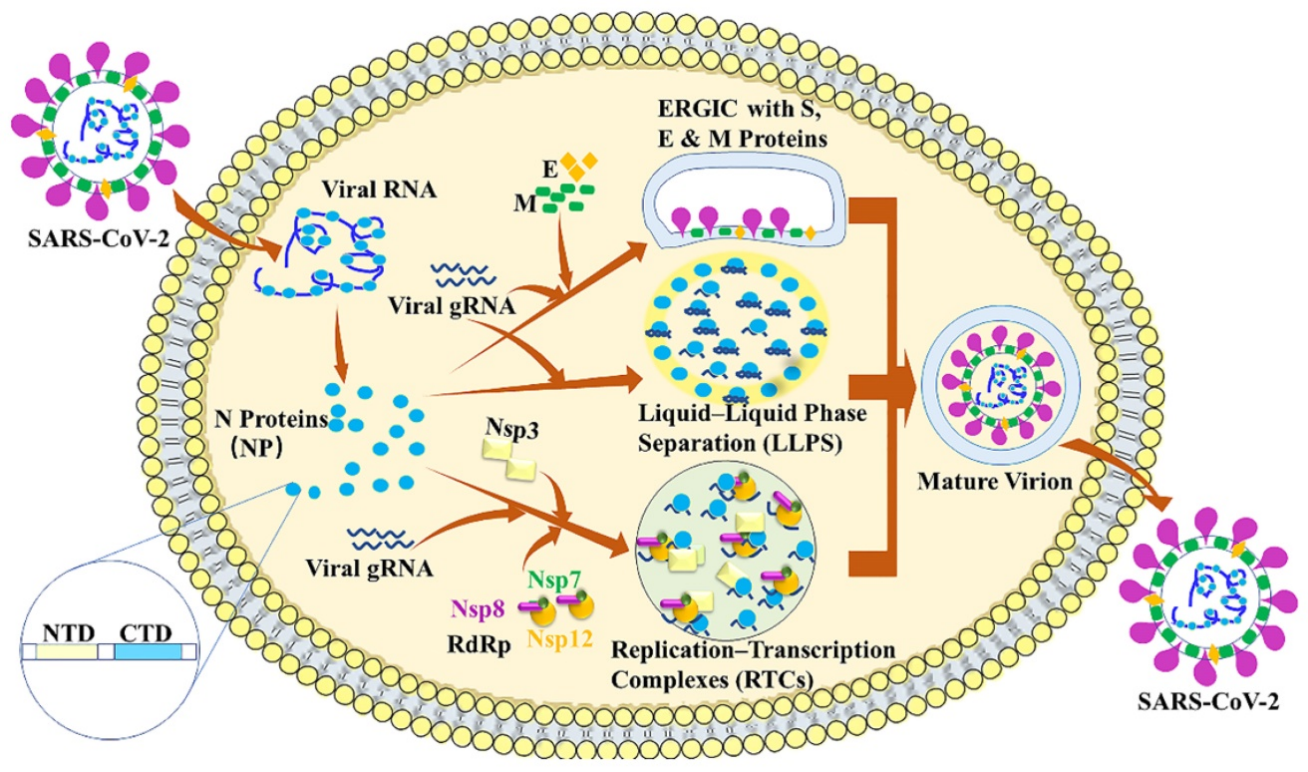
SARS-CoV-2 Nucleocapsid protein. The nucleocapsid (N) protein plays three important roles in SARS-CoV-2 life cycle. The primary role is to assemble with genomic RNA into the viral RNA-protein (vRNP) complex. Second, N protein can undergo liquid-liquid phase separation (LLPS) with viral genome RNA and potentially facilitate viral assembly. Third, N protein can localize to replication transcription complexes (RTCs) at the early stage of infection, where it enhances replication and the transcription of viral RNA by recruiting virus proteins and host factors.

The SARS-CoV-2 nucleocapsid (N) protein is approximately 90% identical with the SARS-CoV N protein 20. N protein is a key player in viral replication, viral genomic RNA (gRNA) packaging into new virions and modulation of host-cell response to infection. N protein has two conserved and independently folded structural domains, called N-terminal RNA binding domain (NTD) which is responsible for the RNA binding and C-terminal dimerization domain (CTD) 21. N protein exhibits three important roles in coronavirus life cycle (Figure 2). First, the primary role is to assemble with genomic RNA into the viral RNA-protein (vRNP) complex 22. Virion formation occurs via the accumulation of the SARS-CoV-2 structural proteins [S, E, M and N] and gRNA at the ER-Golgi intermediate compartment (ERGIC) membrane. The N protein can interact with a luminal domain of the M protein through C-terminal dimerization domain (CTD) ^(247-365)^, which may be essential for mediating the recruitment of N-containing RNPs to the ERGIC membrane 23. Previous study also suggests that the interaction between N protein and the E protein may plays an important role in SARS-CoV-2 assembly 24. Together with N protein, a single strand of SARS-CoV-2 gRNA forms dense, locally ordered ribonucleoprotein (RNP) regions which may be further organized into more complex arrangements 25-27. Second, N protein can undergo liquid-liquid phase separation (LLPS) with viral genome RNA and potentially facilitates the viral assembly. Many RNA-binding proteins have been found to undergo LLPS with RNA to participate the biological and disease processes 28-32. The LLPS is dependent on the length and concentration of ssRNA. What is more, N protein forms typical sphere-like droplets with short ssRNAs, but not solid-like structures with long ssRNAs. The free Zn^2+^ in cytosol is essential for N protein/RNA LLPS 21. It is also reported that viral RNA can induce assembly of the N protein into phase-separated condensates in vitro and pinpoints a ~40 residue region in the central intrinsically disordered region (IDR) with a key role in RNA-driven phase separation 23.

Third, N protein can localize to replication transcription complexes (RTCs) at the early stages of infection, where it enhances replication and the transcription of viral RNA by recruiting host factors 15,33-36. Previous studies have demonstrated that N protein and RNA recruited components of the RNA polymerase (RdRp) complex (Nsp7, Nsp8 and Nsp12) are responsible for replicating viral gRNA 37,38. The phosphorylation of the SR domain of the N protein can decrease the degree of recruitment of RdRp components to condensates 37. The interaction between N and Nsp3 is essential for viral replication. The N-terminal of N has an RNA-binding domain (N-NTD) that can bind the RNA genome, whereas, the C-terminal domain (N-CTD) can interact with the viral Nsp3 39.

## 3. Nucleocapsid protein undergoes Liquid-Liquid Phase Separation (LLPS) and attenuates stress granules

**Figure 3:**
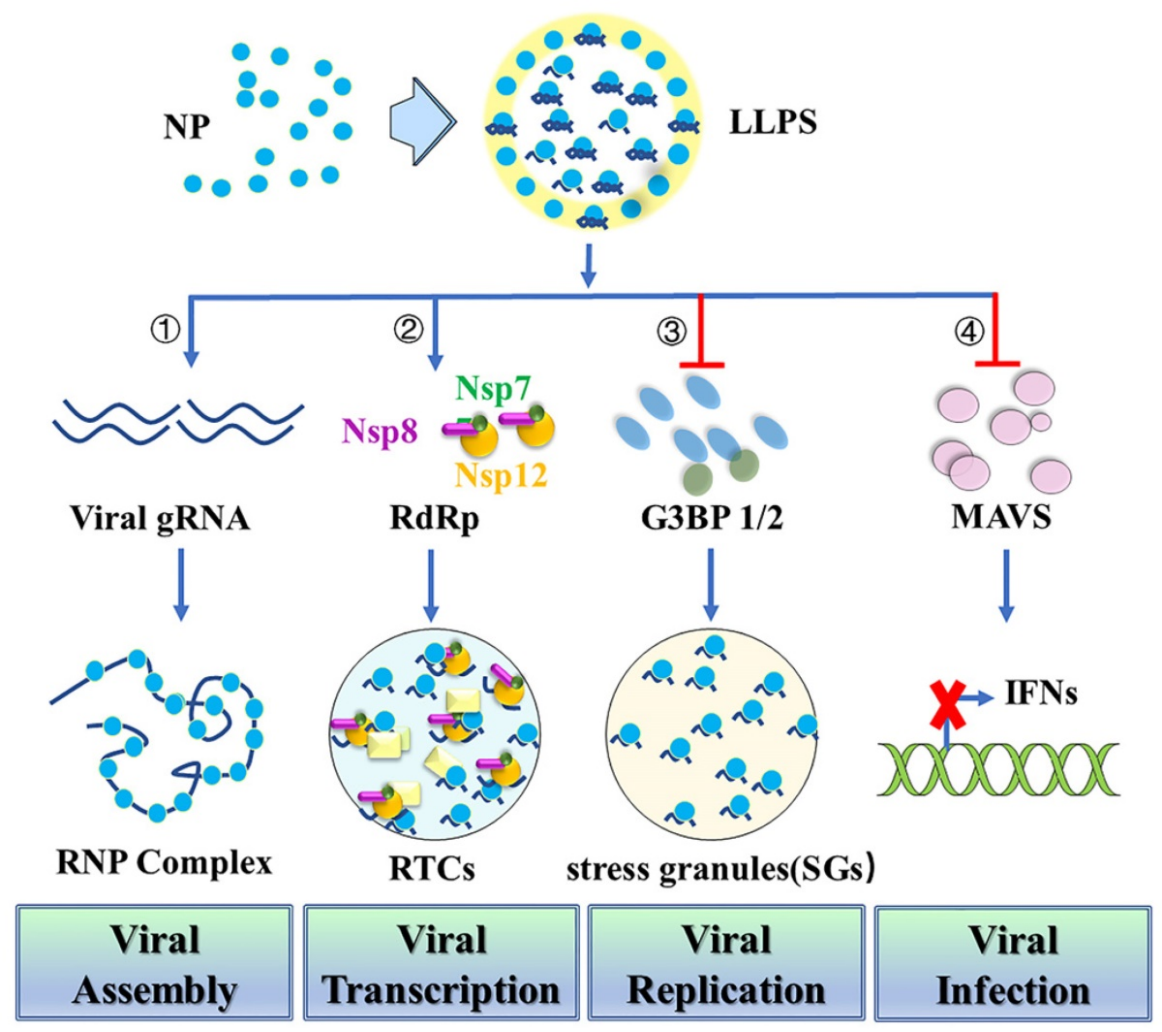
Nucleocapsid protein undergoes Liquid-Liquid Phase Separation (LLPS) and attenuates stress granules. First, N protein can undergo LLPS with viral genome RNA and potentially facilitate viral assembly. Second, N protein LLPS promotes cooperative association of the RdRp complex with polyU RNA to enable high initiation and elongation rates during viral transcription. Third, N protein undergoes LLPS into SGs through its N-terminal intrinsically disordered region (IDR) with SG protein G3BP1/2 to promote viral replication. Fourth, N protein which is required for LLPS with RNA inhibits MAVS and thereby suppresses the innate antiviral immune response to promote viral infection.

LLPS provides a highly cooperative mechanism for proteins and nucleic acids condensation into a dense phase to resemble the liquid droplets 40. During virus infection, LLPS serves as a scaffold for virus replication and promotes the assembly of mature virions through proximity-dependent interactions 41. N protein has sequence and structure features similar to those of other proteins that undergo LLPS with nucleic acids 42. Thus, N protein can undergo LLPS with viral genome RNA to potentially facilitate the viral assembly (Figure 3). Negative staining electronic microscopy (EM) or cryo-EM imaging of N protein/RNA LLPS in the presence and absence of Zn^2+^ reveals similar loose filament-like structures as those observed in the RNP particles of another β-coronavirus MHV 43. This suggests the potential role for N protein/RNA LLPS in viral assembly 21. A recent study also demonstrates that N protein LLPS can promote cooperative association of the RdRp complex with polyU RNA in vitro 37. N protein may also use LLPS-based mechanisms to enable high initiation and elongation rates during viral transcription 37. Phosphorylation of SR-domains of N protein inhibits its RNA binding and RNA-induced LLPS 37. Besides the function in viral assembly and transcription, the dimerization domain of N protein can also inhibit Lys63-linked poly-ubiquitination and aggregation of MAVS, thereby suppressing the innate antiviral immune response 44. N protein acetylation at Lys375 abrogates its LLPS with RNA and the N protein-mediated suppression of MAVS signaling. Targeting the dimerization domain of N protein by a peptide can disrupt the LLPS and then inhibit SARS-CoV-2 replication in vitro and in vivo 44.

N protein can also interact with human ribonucleoproteins which are found in several LLPS-driven cytosolic protein/RNA granules 45. This suggests that N protein may modulate protein/RNA granule formation in order to promote viral replication 46. Stress granules (SGs) are cytoplasmic protein/RNA granules. They are formed through LLPS as an antiviral response to inhibit protein synthesis and induce innate immune signaling 47-49. Recent studies reported that SARS-CoV-2 N protein undergoes LLPS into SGs through its N-terminal intrinsically disordered region (IDR) with SGs protein G3BP1/2 23,50,51. Additionally, N protein inhibits the host stress response through SGs attenuation by sequestering G3BP1/2 through its interaction with these proteins and direct interaction with host mRNAs 51. N protein can also specifically interact with G3BP1/2, leading to a reduction in the size and/or number of stress granules 52-57. A short sequence (residues 15-18) in the IDR NTD or three arginine residues (R92, R107, and R149) in the NTD may also play an important role in its interaction with G3BP1 and modulation of stress granules. Thus, targeting the N-G3BP1 interaction by competitive peptide can reduce viral proliferation, indicating that N-G3BP1 is important for viral replication 52,57. Furthermore, N protein is able to impair SGs formation by inhibiting PKR autophosphorylation and activation, as well as by targeting G3BP1 55.

## 4. Nucleocapsid protein suppresses host pyroptosis but promotes apoptosis

Gasdermin D (GSDMD) is cleaved by the caspase-1 dimers post-inflammasome activation, leading to cell membrane permeability, cell content leakage and finally cell death termed pyroptosis 58,59. While SARS-CoV-2 infection promotes activation of caspase-1 and NLRP3 inflammasome, GSDMD cleavage and pyroptosis are inhibited by N protein in infected human monocytes. N protein binds GSDMD and hinders GSDMD cleavage by the activated caspase-1 dimers to antagonize pyroptosis.

Apoptosis is a major type of programmed cell death. It is triggered by mitochondrion or cell-surface death receptor mediated the cleavage of downstream caspases 60-62. Previous studies indicated that SARS-CoV N protein can induce apoptosis in HPF cells and COS-1 cells by activating the mitochondrial pathway 63-65. Recently study also demonstrated that SARS-CoV-2 N protein can specifically enhance the M protein-induced apoptosis by strengthening M protein-mediated attenuation of PDK1-PKB/Akt interaction 66. Our recent study also reveals that SARS-COV-2 N protein not only promotes renal cell death in ischemic-induced AKI but inhibits renal tubular cell proliferation via Smad3-p21 dependent G1 cell cycle arrest 67. We further uncover that genetic deletion of Smad3 or pharmacological inhibition of Smad3 can protect kidneys from SARS-CoV-2 N protein-induced renal cell death. In addition, SARS-COV-2 N can interact with α-synuclein to disturb the α-synuclein proteostasis and increase cell death in S-SY5Y 68. This may provide molecular basis for the correlation between SARS-COV-2 infections and Parkinsonism.

## 5. Regulation of host inflammation by Nucleocapsid protein

**Figure 4:**
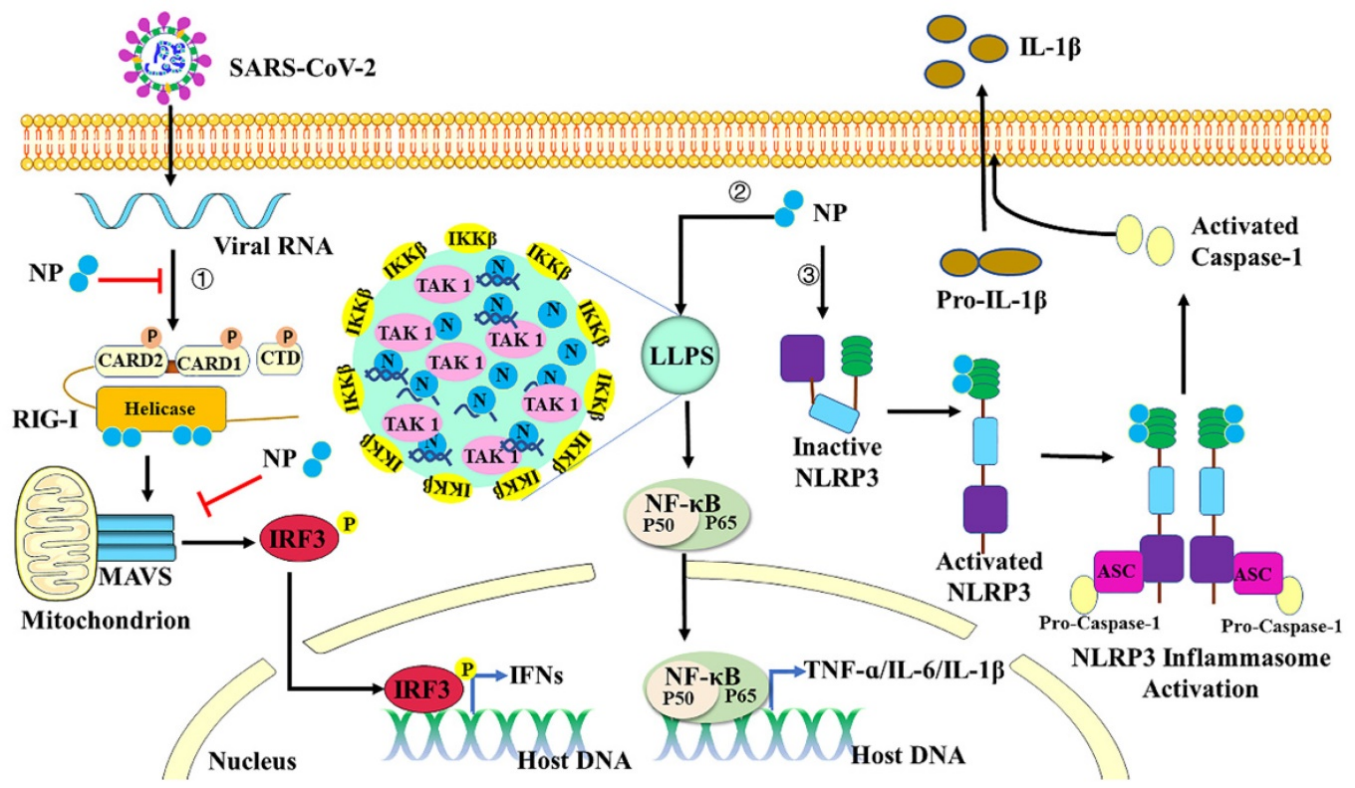
Regulation of host inflammation by Nucleocapsid protein. First, SARS-COV-2 N protein can inhibit IFN-β production by targeting each step of the RIG-I signaling pathway. Second, with viral RNA, N protein undergoes LLPS to recruit TAK1 and IKK complex, and then promotes NF-κB activation. Third, N protein interacts directly with NLRP3 protein to promote the assembly and activation of the NLRP3 inflammasome.

Host immune response including innate and adaptive immunity against SARS-CoV-2 seems crucial to control and resolve the viral infection 69-71. The innate immune system detects viral infections through the recognition of molecular patterns. It is a primary host defense strategy to suppress viral infections, coordinate and accelerate the development of adaptive immunity 72. Pattern recognition receptors (PRRs) respond to pathogen-associated molecular patterns (PAMPs) can trigger the activation of inflammatory responses and the release of inflammatory cytokines to limit viral infection 73. Several families of PRRs have been described: the Toll-like receptor (TLR) 74, the RIG-I-like receptor (RLR) 75, the NOD-like receptor (NLR), and the C-type lectin receptor (CLR) 76. Single-stranded RNA derived from genomic, subgenomic or replicative intermediates of SARS-CoV-2 can be sensed by RLRs, which include MAD5, RIG-I and LGP2 77-80. RIG-I and MDA5 are the most well-studied RLRs and critically regulate IFN pathways. After virus infection, RIG-I and MDA5 sense the RNA and then translocate to the mitochondria, where they interact with the adaptor protein mitochondrial antiviral signaling (MAVS) to form a MAVS signalosome. This complex formation activates downstream proteins to induce phosphorylation of IRF3, therefore facilitating its nuclear translocation and the transcription of genes encoding type I and III IFNs 81,82. Production and subsequent release of IFNs can stimulate the downstream signals to produce hundreds of IFN-stimulated genes (ISGs) with various antiviral functions 83,84. Previous studies demonstrated that coronaviruses (CoVs) have evolved evasion strategies to limit host control. Otherwise, they enhance replication and transmission in response to innate immune-dependent viral clearance mechanisms 69,85-87. In SARS-COV-2 infection, a low production of type I interferons was detected in the peripheral blood or lungs of COVID-19 patients with a severe clinical picture 69,88,89. In a recent study, SARS-COV-2 N protein can interact with TRIM25 functional domain SPRY to block the ubiquitinating activity of TRIM25 on RIG-I and then inhibit IFN-β production 90. SARS-COV-2 N protein can also interact with G3BP1 to prevent the antiviral stress granule formation and impair the recognition of dsRNA by RIG-I 91. It is also reported that SARS-COV-2 N protein is able to interact with RIG-I through the DExD/H domain of RIG-I and then to repress RIG-I-mediated IRF3 phosphorylation and nuclear translocation. N protein suppresses IFN-β production upon the infection of SeV or by the stimulation of poly(I:C) 92. In addition, N protein is found to inhibit Lys63-linked poly-ubiquitination and aggregation of MAVS, thereby suppressing the innate antiviral immune response 44. All these results indicate that SARS-COV-2 N protein can inhibit IFN-β production by targeting the RIG-I signaling pathway (Figure 4).

In most cases, innate immune responses limit viral entry, translation, replication, and assembly. However, in some individuals, the disease severity or mortality of the COVID-19 might be associated with the excessive production of proinflammatory cytokines, resulting in “cytokine storm” and acute respiratory distress syndrome 93. Nuclear factor κB (NF-κB) is a key transcription factor of proinflammatory cytokines in immune cells 94. Upon sensing different ligands, their receptors then recruit the adaptor proteins to promote the activation of tumor necrosis factor receptor (TNF-R)-associated factor (TRAF) signaling molecules, thus recruiting TGF-beta-activated kinase 1 (TAK1) and IκB kinase (IKK) complex 95. Activated IKK complex can phosphorylate IκB proteins to induce ubiquitin-proteasome degradation. Degradation of IκB allows NF-κB translocation to the nucleus to initiate the transcription of downstream proinflammatory cytokines 96. SARS-CoV-2 N protein is reported to promote activation of NF-κB signaling by enhancing the association between TAK1 and IKK complex 95. With viral RNA, N protein undergoes LLPS to recruit TAK1 and IKK complex, and then to promote NF-κB activation. The CTD domain of SARS-CoV-2 N protein is critical for its LLPS and NF-κB activation. 1,6-hexanediol which is the inhibitor of LLPS can inhibit the phase separation of N protein and then suppress the activation of NF-κB. All these results indicate that LLPS of N protein provides a platform to induce NF-κB activation after virus infection. SARS-CoV-2 N can also function as a PAMP to directly bind to TLR2 to activate NF-κB and MAPK signaling in endothelial cells 97. Treatment with recombinant SARS-CoV-2 N protein can induce acute lung injury via M1 macrophage polarization and NF-κB activation, which can be inhibited by N-protein denaturation, neutralizing antibody to N-protein, and NF-κB inhibitor 98. NLRs are also reported to respond to SARS-CoV-2 infection and induce production of pro-inflammatory cytokines 99. NLRP3, one of the best characterized inflammasome sensors, is triggered in response to virus infection and thus activates Caspase-1 with an adaptor protein ASC 100. Active caspase-1 is formed by autocatalytic cleavage, which then catalyzes proteolytic processing of pro-interleukin (IL)-1β and pro-interleukin (IL)-18 into mature IL-1β and IL-18 101. IL-1β plays crucial roles in inflammatory responses and instructs adaptive immune responses by inducing expression of immunity associated genes 102. SARS-CoV-2 N protein is reported to induce proinflammatory cytokines through promoting the assembly and activation of the NLRP3 inflammasome 100. Indeed, N protein interacts directly with NLRP3, promotes the binding of NLRP3 with ASC, and facilitates NLRP3 inflammasome assembly. More importantly, N protein aggravates lung injury and accelerates death in acute inflammation mouse models, which can be blocked by NLRP3 inhibitor MCC950 and Caspase-1 inhibitor Ac-YVAD-cmk. Taken together, SARS-CoV-2 N protein induces proinflammatory cytokines through promoting the activation of NF-κB signaling and NLRP3 inflammasome (Figure 4).

RNAi, a post-transcriptional gene silencing mechanism, has been recognized as an antiviral immune defense after virus infection 103. After virus infection and replication, virus-derived dsRNA is generated and can be recognized and cleaved by the host endoribonuclease Dicer. As a countermeasure, viruses such as Influenza A virus NS1 and Dengue virus 2 NS2A can encode viral protein to inhibit the RNAi pathway 103,104. Recently study also reported that SARS-CoV-2 N protein can suppress RNAi pathway 105. Indeed, N protein can interact with dsRNA and then sequestrates dsRNA to suppress RNAi, thereby functioning as a key immune evasion factor of SARS-CoV-2.

Based on the function of SARS-CoV-2 N protein, we proposed a working model for SARS-CoV-2 induced inhibition of host innate immunity. In the early stage of infection, N protein can interact with TRIM25 or G3BP1 to influence RIG-I activation. N protein can also interact with DExD/H domain of RIG-I directly and inhibit RIG-I activation. What is more, N protein is capable of interacting with an adaptor protein MAVS to inhibit the ubiquitination of MAVS. Taken together, N protein represses RIG-I-mediated phosphorylation of TBK1 and IRF3 to suppress their nuclear translocation and IFN-β expression. At the same time, N protein can also suppress RNAi to evade the innate immune system. However, at the later stage of infection, the viral replication, transcription, and assembly are actively processed with the involvement of various viral proteins and inflammatory pathways, which may trigger the overactivation of innate immunity, resulting in cytokine storm syndrome and disease progression.

## 6. Conclusions and perspectives

Here, we summarize the signaling mechanisms of SARS-CoV-2 N protein in viral replication, cell death, and inflammation. N protein as a structure protein plays a critical role in multiple steps of the viral life cycle. N protein assembles with genomic RNA into the viral RNA-protein (vRNP) complex to facilitate viral assembly. Moreover, it contributes to forming helical ribonucleoprotein during the packaging of the RNA genome and regulating the viral RNA synthesis during replication and transcription. Importantly, N protein has multiple functions in cell death and inflammation. N protein can inhibit pyroptosis but promotes apoptosis to induce cell death. N protein can inhibit RIG-I and RNAi signaling but promotes NF-κB signaling and NLRP3 inflammasome in the innate immune system to trigger the “cytokine storm”.

Recent phosphoproteomic analyses revealed that SARS-CoV-2 N protein is highly phosphorylated within the RS domain 106-109. It is known that phosphorylation regulates the states and functions of the N protein. Phosphorylation of N protein by glycogen synthase kinase 3(GSK-3) is required for viral transcription and replication 110. GSK-3 is essential for SARS-CoV-2 N phosphorylation as blockade of GSK-3 with inhibitors can block N phosphorylation and virus replication in SARS-CoV-2 infected lung epithelial cells 111. Thus, research into a better understanding of the phosphorylation of SARS-CoV-2 N protein in viral transcription and replication is needed.

Based on the high homology (90%) of the amino acid sequences and fewer mutations over time among coronavirus N proteins 112, SARS-CoV-2 N protein may function similarly to SARS-CoV N or MERS-CoV N protein. The SARS-CoV N protein has been reported to interact with numerous host cell proteins, such as TRIM25 113, Smad3 114, the chemokine Cxcl16 115, translation elongation factor-1 alpha 116, pyruvate kinase 117, and 14-3-3 118. Although SARS-CoV-2 N can also interact with TRIM25 90, Smad3 67,119, 14-3-3 120, and others 121, further research into the interaction between SARS-CoV 2 N and host cell proteins under disease conditions may provide valuable information for potential druggable targets. Thus, understanding of the roles and mechanisms of N protein in the pathogenesis of diseases may be the first step towards the development of anti-SARS-CoV-2 drugs and vaccines to prevent and control the SARS-CoV-2 pandemics.

## Abbreviations

ARDS: acute respiratory distress syndrome
ASC: apoptosis-associated speck-like protein with the CARD domain
COVID-19: coronavirus disease 2019
CLR: C-type lectin receptor
CTD: C-terminal dimerization domain
ERGIC: ER-to-Golgi intermediate compartment
IDR: intrinsically disordered region
RIG-I: retinoic-acid inducible gene-I
MDA5: melanoma differentiation-associated protein 5
IKK: IκB kinase
LLPS: liquid-liquid phase separation
MAVS: mitochondrial antiviral signaling
N: Nucleocapsid
NF-κB: Nuclear factor κB
NLR: NOD-like receptor
Nsps: nonstructural proteins
NTD: N-terminal RNA binding domain
ORFs: open reading frames
PAMPs: pathogen-associated molecular patterns
RdRp: RNA dependent RNA polymerase
RLR: RIG-I-like receptor
PRRs: Pattern recognition receptors
RTCs: replication transcription complexes
SARS-CoV-2: severe acute respiratory syndrome coronavirus 2
SGs: Stress granules
TAK1: TGF-beta-activated kinase 1
TLR: Toll-like receptor
TRAF: tumor necrosis factor receptor (TNF-R)-associated factor
vRNP: viral RNA-protein
WHO: World Health Organization
